# Assessment of MALDI-TOF MS as Alternative Tool for *Streptococcus suis* Identification

**DOI:** 10.3389/fpubh.2015.00202

**Published:** 2015-08-21

**Authors:** Marta Pérez-Sancho, Ana Isabel Vela, Teresa García-Seco, Marcelo Gottschalk, Lucas Domínguez, José Francisco Fernández-Garayzábal

**Affiliations:** ^1^Centro de Vigilancia Sanitaria Veterinaria (VISAVET), Universidad Complutense, Madrid, Spain; ^2^Campus de Excelencia Internacional (CEI) Moncloa, Universidad Politécnica de Madrid (UPM), Universidad Complutense de Madrid (UCM), Madrid, Spain; ^3^Departamento de Sanidad Animal, Facultad de Veterinaria, Universidad Complutense, Madrid, Spain; ^4^Groupe de Recherche sur les Maladies Infectieuses du Porc, Faculté de Médecine Vétérinaire, Université de Montréal, Saint-Hyacinthe, QC, Canada

**Keywords:** identification, MALDI-TOF MS, *Streptococcus suis*, PCR, biochemical tests

## Abstract

The accuracy of matrix-assisted laser desorption ionization time-of-flight mass spectrometry (MALDI-TOF MS) for identifying *Streptococcus suis* isolates obtained from pigs, wild animals, and humans was evaluated using a PCR-based identification assay as the gold standard. In addition, MALDI-TOF MS was compared with the commercial multi-tests Rapid ID 32 STREP system. From the 129 *S. suis* isolates included in the study and identified by the molecular method, only 31 isolates (24.03%) had score values ≥2.300 and 79 isolates (61.24%) gave score values between 2.299 and 2.000. After updating the currently available *S. suis* MALDI Biotyper database with the spectra of three additional clinical isolates of serotypes 2, 7, and 9, most isolates had statistically significant higher score values (mean score: 2.65) than those obtained using the original database (mean score: 2.182). Considering the results of the present study, we suggest using a less restrictive threshold score of ≥2.000 for reliable species identification of *S. suis*. According to this cut-off value, a total of 125 *S. suis* isolates (96.9%) were correctly identified using the updated database. These data indicate an excellent performance of MALDI-TOF MS for the identification of *S. suis*.

## Introduction

*Streptococcus suis* is one of the most important pathogens in the swine industry worldwide, causing meningitis and a wide range of diseases, such as, arthritis, endocarditis, pneumonia, and septicemia (1). Furthermore, *S. suis* has been isolated from a range of other mammalian and avian species (2–4). *S. suis* has also been recognized as an emerging human pathogen over the past few years, affecting people in close contact with pigs or pork-derived products (3).

The clinical significance of *S. suis* infections in both human and animal medicine makes necessary to have diagnostic tools able to accurately identify this pathogen. Despite PCR assays for the detection and identification of *S. suis* have been developed (5–7), in many diagnostic laboratories, the identification of this pathogen is still based on bacteriological and biochemical criteria, mainly using commercial multi-test systems that, in general, present controversial results (8). Veterinary diagnostic laboratories can easily identify *S. suis* isolates from clinical cases in pigs, but identification of *S. suis* from healthy pigs can be more difficult due to the presence of other streptococci that are part of the normal tonsillar microflora and are phenotypically similar to *S. suis* (8). Human diagnostic laboratories can also misidentify *S. suis* with other microorganisms, such as, enterococci, *S. bovis* or viridans group streptococci (8, 9). More recently, MALDI-TOF MS has emerged as a reliable high-throughput tool for microbiological identification. This technique has been demonstrated as a reliable alternative tool for identification of gram-positive bacteria (10), including *Streptococcus* spp. isolates (11–15). However, particular identification of *S. suis* using this approach has not yet been thoroughly reported although this technique could overcome the drawbacks of current routine identification techniques and may contribute to a better understanding of its impact in animal production and public health.

Hence, this study was conducted to evaluate the accuracy of matrix-assisted laser desorption ionization time-of-flight mass spectrometry (MALDI-TOF MS) for identifying *S. suis* isolates obtained from pigs, wild animals and humans, using a polymerase chain reaction (PCR)-based identification as the gold standard. In addition, MALDI-TOF MS was compared with the commercial multi-tests Rapid ID 32 STREP system.

## Materials and Methods

### Bacterial strains

Overall, 129 *S. suis* isolates were used in the study. These include 62 and 64 isolates recovered from swine and wild animals (wild boar, wild rabbit, and Iberian wild goat), respectively, belonging to different serotypes (2 *n* = 25; 7 *n* = 5; 9 *n* = 69; less prevalent serotypes *n* = 27). Pig isolates were recovered from Spain (*n* = 47) or Canada (*n* = 15), while wild animal isolates were all from Spain. All animal isolates from Spain were obtained from the culture collection of VISAVET Centre (Universidad Complutense de Madrid). Three human serotype 2 isolates were also included in the study. Identification of all isolates was confirmed by a species-specific PCR assay described by Okwumabua et al. (6) which have been considered the gold standard technique in this work. All isolates were grown overnight on Columbia sheep agar plates at 37°C under aerobic conditions.

Isolates with different identification levels based in the Rapid ID 32 STREP system (bioMérieux) were included in order to assess the capability of MALDI-TOF MS technology to overcome the drawbacks detected in biochemical identification. Rapid ID 32 STREP identifications were categorized as follows: species identification (which included acceptable, good, very good, and excellent identification to species level), genus identification (including doubtful identification to species level or acceptable and good identification to genus level), and unreliable identification (which contains unacceptable identification or identified as non-*S. suis*).

### MALDI-TOF MS analysis

Spectra from each isolate were obtained after ethanol formic acid extraction in accordance with manufacturer’s instructions using fresh and pure cultures considered an efficient sample preparation method for gram-positive bacteria (16, 17). One microliter of each isolate extract was spotted onto a 384-spot polished steel target plate, let to dry at room temperature and overlaid with one microliter of α-cyano-4-hydroxy-cinnamic acid (HCCA) matrix. Data acquisition was performed using a Bruker Daltonics UltrafleXtrem MALDI TOF/TOF equipment and the Biotyper Real Time Classification software v3.1 (Bruker Daltonics, Bremen, Germany).

In order to improve the already available *S. suis* MALDI Biotyper database (including five entries) and generate an updated database (UDB), the MALDI-TOF MS analysis of three additional clinical isolates of serotypes 2, 7, and 9 were performed. The 24 spectra obtained from the eight spots for each of the three strains were analyzed by FlexAnalysis (version 3.0, Bruker Daltonics) according to Rettinger et al. (18). A minimum of 20 accurate spectra were downloaded in MALDI Biotyper (version 3.0, Bruker Daltonics) to create a main spectrum profile (MSP) of each strain according to the manufacturer’s suggestions.

### MALDI-TOF MS identification

All 129 isolates were analyzed by MALDI-TOF MS as described above and the spectra obtained were compared with the original MALDI Biotyper database (ODB) and the UDB. The reliability of the identification using MALDI Biotyper was performed according to the log (score) values calculated by the Biotyper software (version 3.1; 4613 entries) according to manufacturer’s parameters: highly probable identification at species level: scores of ≥2.300; secure genus and probable species identification: scores between 2.299 and 2.000; probable genus level identification: score of 1.999–1.700; unreliable identification: scores <1.700. For each isolate with scores ≤2.000, MALDI-TOF MS analysis and extraction protocol was repeated at least in two independent runs.

### Statistical analysis

Agreement between the results obtained with both techniques was assessed applying the kappa test. Proportions of positive samples were compared using *Z*-test (adjust *p*-values – Bonferroni methods). To evaluate a possible association between MALDI quantitative results (scores) and the categorized API profiles, ANOVA test was used. In addition, the comparison of the usefulness of the ODB and UDB were assessed using the Paired-sample *t*-*t*est. Analysis of the data was carried out using software SPSS 20 (Statistical Package for the Social Sciences, IBM, New York, USA).

## Results

A total of 31 isolates (24.03%; mean score = 2.348) gave scores values ≥2.300, another 79 isolates (61.24%; mean scores = 2.188) gave score values between 2.299 and 2.000 and 19 isolates (14.7%) had score values between 1.999 and 1.748 (Table 1). Except in one isolate identified as *Streptococcus pneumoniae* (score value of 1.748), the first identification option by MALDI-TOF MS, irrespective of the score value, was always *S. suis*.

**Table 1:** **Comparison of MALDI-TOF MS results depending on database and threshold score used in a panel of 129 *S. suis* isolates recovered from swine, wild animals, and humans**.

Identification level	MALDI-TOF MS		
	ODB^a^ No. (%)	UDB^b^	
		Bruker’s threshold score	Proposed threshold score
		No. (%)^c^	No. (%)^d^
Highly probable specie	31 (24)	106 (82.2)	125 (96.90)
Probable specie, secure genus	79 (61.2)	19 (14.73)	4 (3.1)
Probable genus	19 (14.7)	4 (3.1)	0 (0)

*^a^ODB: original database of MALDI Biotyper (4613 entries with 5 *S. suis* entries; Bruker Daltonics, Germany). Bruker’s threshold identification scores: highly probable species identification: scores of ≥2.300; secure genus and probable species identification: scores between 2.299 and 2.000; probable genus level identification: score of 1.999–1.700; unreliable identification: scores <1.700*.

*^b^UDB: updated database (after inclusion of three new *S. suis* entries of serotypes 2, 7, and 9)*.

*^c^Same Bruker’s threshold identification scores as ODB*.

*^d^Reliable (highly probable and probable) species identification: scores of ≥2.000; secure genus level identification: scores between 1.999 and 1.700; unreliable identification: scores <1.700*.

There were differences on the percentage of isolates identified at species level with a score value ≥2.300 (highly probable species) depending on serotype. Thus, the 42.9% (12/28), 20% (1/5), and 18.8% (13/69) of isolates of serotypes 2, 7, and 9, respectively, had score values ≥2.300. These percentages were not significantly higher for any of the analyzed serotypes (*Z*-test, adjust *p*-values – Bonferroni methods). The percentage isolates belonging to less prevalent serotypes with score values ≥2.300 was 19.2% (5/26; Table 2). Regarding the host from which *S. suis* was recovery, no significant differences (*Z*-test, adjust *p*-values – Bonferroni methods) were observed in the percentage of pig (18/62, 29%) and wild animals (13/63, 20.63%) isolates identified as species level with a score value ≥2.300 by MALDI-TOF MS.

**Table 2:** **Percentage of *S. suis* isolates with score values ≥2.300 based on serotype with the original Bruker’s MALDI-TOF MS database (ODB; including 5 *S. suis* entries) and the updated database (UDB; after inclusion of three new *S. suis* entries)**.

Serotype	%ODB (*N*/total)	%UDB (*N*/total)	Fold-increase
2	42.9 (12/28)	89.3 (25/28)	2.1
7	20 (1/5)	100 (5/5)	5
9	18.8 (13/69)	88.4 (61/69)	4.7
Less prevalent	19.2 (5/26)	55.6 (15/27)	2.9

The agreement between Rapid ID 32 STREP and MALDI-TOF MS results (considering isolates with score values of at least 2.300) was slight (κ = 0.035) in the present study (*n* = 129). Comparison of the MALDI-TOF MS quantitative results (score values) of the three API ID 32 STREP identification categories revealed that those isolates classified as unreliable identification (unacceptable identification or misidentified by the Rapid ID 32 STREP strips) gave significantly lower score values (ANOVA test, *p* < 0.05) than those isolates included in the other two identification categories (identification at species and genus level).

To improve identification of *S. suis*, we constructed spectra containing information on peak masses and peak intensities of three clinical isolates of serotypes 2, 7, and 9, which were used to improve the original reference database (ODB) and create an UDB. After the addition of the three new *S. suis* profiles, most isolates presented statistically significant higher score values (Paired-sample *t*-test, *p* < 0.001) when their spectra were matched with the UDB (mean score: 2.65) than those obtained using the ODB (mean score: 2.182). These higher score values resulted in an increase in the number (106; 82.2%) of *S. suis* isolates with scores values ≥2.300 (Table 1). Considering serotype, the 89.3% (25/28), 100% (5/5), 88.4% (61/69) and 55.6% (15/27) of isolates of serotypes 2, 7, 9 and less prevalent serotypes, respectively, had score values ≥2.300 (Table 2). The agreement between Rapid ID 32 STREP and MALDI-TOF MS results slightly increased (*κ* = 0.286) when the spectra of *S. suis* isolates were matched against the UDB database.

**Figure 1:**
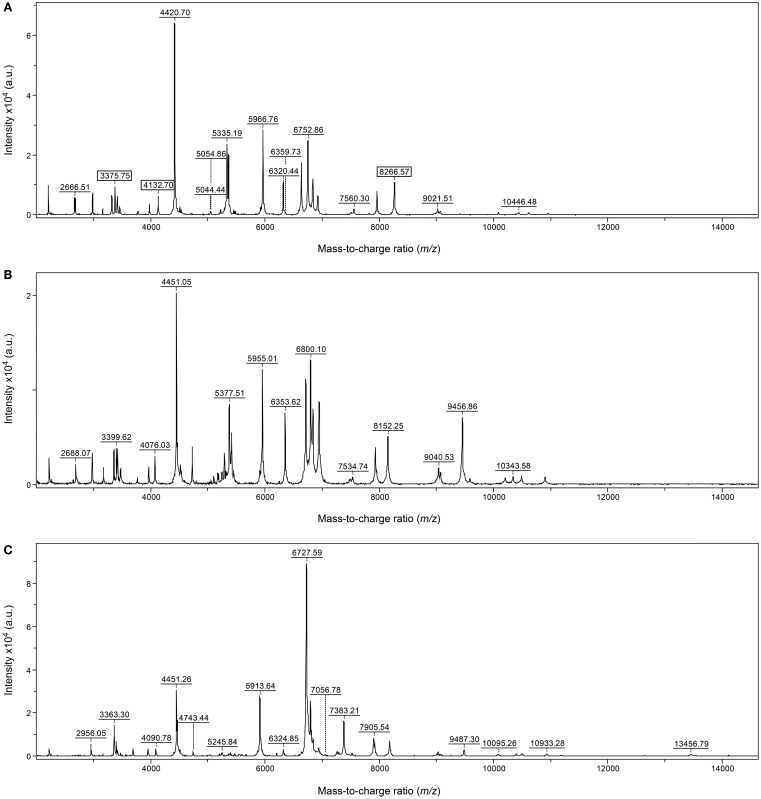
**MALDI-TOF MS mass spectra of *S. suis* (A), *S. porcinus* (B), and *S. dysgalactiae* subsp. *equisimilis* (C)**. The *y* axis represents the relative intensities (%) and the *x* axis represents the *m*/*z* values (masses in Da).

None of the *S. suis* isolates included in the present study was misidentified with *Streptococcus porcinus* and *Streptococcus dysgalactiae* subsp. *equisimilis*, the other two most important pathogens of swine belonging to this genus, included in the ODB. The spectra of *S. suis* presented, regardless their serotype, distinct MS peaks (e.g., *m*/*z* 3377, 4133, and 8267) which allowed its differentiation from these two streptococcal species by MALDI-TOF MS technique (Figure 1).

## Discussion

MALDI-TOF MS is a powerful tool that has attracted worldwide attention for its direct and rapid discrimination and identification of different microorganisms. In the present work, the performance of MALDI-TOF MS system for *S. suis* identification was evaluated using as gold standard a species-specific PCR [this system has been widely used for reliable identification of *S. suis* (5, 19–22)]. These results were compared with the identification results obtained previously using the commercial identification system Rapid ID 32 Strep (bioMérieux). Using the threshold score established by the manufacturer for highly probable species identification (score ≥ 2.300), the MALDI-TOF MS system was able to identify only a quarter (24.03%) of the *S. suis* isolates. This percentage is relatively low compared with the 63.6% of *S. suis* isolates (*n* = 82) that gave acceptable to excellent identification results using the commercial Rapid ID 32 STREP strips. The 31 *S. suis* isolates properly identified by MALDI-TOF MS were also correctly identified by the Rapid ID 32 STREP system, while the remaining 51 isolates correctly identified by this last system gave score values between 2.000 and 2.299 (considered probable species identification).

Serotype 2 includes most of the clinical pig isolates worldwide (9), but other serotypes, such as, serotypes 7 or 9 are also epidemiologically relevant (8). Serotype 2 showed a higher percentage of isolates with score values ≥2.300 (12/28; 42.85%) by MALDI-TOF MS system than serotypes 9 (13/69; 18.84%) and 7 (1/5; 20%) (Table 2). The relatively low performance of MALDI-TOF MS system for the identification of isolates of the latter serotypes might represent a limitation for the routinely identification of this pathogen.

As the quality and reliability of the identification by MALDI-TOF MS depends on the quality and amount of reference spectra present in the database, the original Bruker’s reference database (ODB) was implemented by adding the spectra obtained from three additional clinical isolates of serotypes 2, 7, and 9, creating an updated reference database (UDB). After the implementation of the database, the MALDI TOF analysis of the 129 strains of *S. suis* used in this study resulted in an increase in the number of isolates (from 24 to 82.2%; Table 1) with scores values ≥2.300. This increase was observed in all serotypes but was more evident in isolates of serotypes 7 and 9 in which the percentage of isolates accurately identified increased 5- and 4.7-fold times, respectively (Table 2). These results highlight the importance of the implementation of MALDI-TOF MS database to improve the discriminatory power of this identification approach, especially in bacteria with high genetic heterogeneity as *S. suis* (23) which could exhibit high diverse protein profiles.

Previous studies have suggested the convenience of using less stringent cut-off values to improve the accuracy of MALDI-TOF MS at the species level (24–26). Considering the PCR and MALDI-TOF results of the present study in which all but one of the 129 isolates in our study (99.2%) were identified as *S. suis* by MALDI-TOF MS as first option irrespective of their score values, we suggest using a less restrictive threshold score of ≥2.000 for a reliable species identification of *S. suis*. According to this cut-off value, a total of 125 isolates (96.9%) were correctly identified as *S. suis* (Table 1) using the UDB, indicating an excellent performance of MALDI-TOF MS.

MALDI TOF system was able to discriminate between *S. suis* and *S. porcinus* and *S. dysgalactiae* subsp. *equisimilis* profiles included in the Biotyper Database. In fact, these bacterial species can also be frequently isolated from diseased-piglets (1). On the other hand, *Streptococcus plurextorum*, *Streptococcus porci* and *Streptococcus porcorum* have been isolated from pigs in the last years (27–29). These species are phylogenetically close related to *S. suis*, but their type strains are not yet available in the MALDI Biotyper database. Therefore, further investigations are required to compare the spectra profiles among these closely related streptococci.

In summary, MALDI-TOF MS represent a rapid, accurate and cost-saving method and a reliable alternative to PCR-based methods for routinely identification of *S. suis* isolates from both human and animal origins.

## Conflict of Interest Statement

The authors declare that the research was conducted in the absence of any commercial or financial relationships that could be construed as a potential conflict of interest. The Guest Associate Editor Andres M. Perez declares that, despite having collaborated with the author Lucas Dominguez, the review process was handled objectively and no conflict of interest exists.
